# What are the essential determinants of human papillomavirus carcinogenesis?

**DOI:** 10.1128/mbio.00462-24

**Published:** 2024-10-04

**Authors:** Karl Munger, Elizabeth A. White

**Affiliations:** 1 Department of Developmental, Molecular and Chemical Biology, Tufts University School of Medicine, Boston, Massachusetts, USA; 2 Department of Otorhinolaryngology: Head and Neck Surgery, University of Pennsylvania Perelman School of Medicine, Philadelphia, Pennsylvania, USA; Albert Einstein College of Medicine, Bronx, New York, USA

**Keywords:** papillomavirus, carcinogenesis, host-cell interactions, transformation

## Abstract

Human papillomavirus (HPV) infection is the leading viral cause of cancer. Over the past several decades, research on HPVs has provided remarkable insight into human cell biology and into the pathology of viral and non-viral cancers. The HPV E6 and E7 proteins engage host cellular proteins to establish an environment in infected cells that is conducive to virus replication. They rewire host cell signaling pathways to promote proliferation, inhibit differentiation, and limit cell death. The activity of the “high-risk” HPV E6 and E7 proteins is so potent that their dysregulated expression is sufficient to drive the initiation and maintenance of HPV-associated cancers. Consequently, intensive research efforts have aimed to identify the host cell targets of E6 and E7, in part with the idea that some or all of the virus-host interactions would be essential cancer drivers. These efforts have identified a large number of potential binding partners of each oncoprotein. However, over the same time period, parallel research has revealed that a relatively small number of genetic mutations drive carcinogenesis in most non-viral cancers. We therefore propose that a high-priority goal is to identify which of the many targets of E6 and E7 are critical drivers of HPV carcinogenesis. By identifying the cancer-driving targets of E6 and E7, it should be possible to better understand the distinct roles of other targets, perhaps in the viral life cycle, and to focus efforts to develop anti-cancer therapies on the subset of virus-host interactions for which therapeutic intervention would have the greatest impact.

## CANCER IS A RARE OUTCOME OF HIGH-RISK HPV INFECTION

Human papillomaviruses (HPVs) are a large family of viruses with small, circular, double-stranded DNA genomes of approximately 8,000 base pairs (bp). Genome sequence diversity is a hallmark of HPVs, and over 400 distinct virus genotypes have been identified. HPVs have a shared tropism for keratinocytes, which are the major constituent of stratified squamous epithelia, and most HPVs share a common genome organization and encode a similar complement of viral genes (1). Diverse HPVs also share a replication strategy that is closely linked to keratinocyte differentiation (2). Long-term persistent HPV infection initiates and is maintained in proliferative basal keratinocytes. Subsequent keratinocyte differentiation enables viral genome replication to a high copy number and the production of infectious virions (3). Although several features of HPV biology are shared among many virus genotypes, the viruses differ in their propensity to infect mucosal or cutaneous epithelia, in the host cell proteins that are targeted by specific HPV-encoded proteins, and in the wide range of pathologies that are associated with HPV infection (1).

Infection with most HPV genotypes causes benign or clinically inapparent lesions, but about a dozen HPVs, including HPV16 and HPV18, are the mucosotropic “high-risk” genotypes that cause lesions that can progress to cancer (4). Even though cancer is a rare outcome of high-risk HPV infection, the infections are so frequent that they cause at least 5% of all human cancer cases (5). The mechanisms by which HPV infection initiates or drives carcinogenesis are directly related to features of the HPV life cycle. Persistent HPV infection likely disrupts the normal balance between proliferation and differentiation in infected basal keratinocytes, biasing cells to be maintained in the proliferative basal epithelial compartment and limiting their commitment to differentiation (2). Subsequent HPV DNA replication in suprabasal keratinocytes requires the virus to drive cell cycle re-entry in otherwise terminally differentiated, growth-arrested cells. Unscheduled cell cycle progression triggers pro-apoptotic signals that must be silenced to preserve the viability of infected cells. Nonetheless, even though many of the host cell reprogramming events that are required for HPV replication have oncogenic potential, they are tightly regulated during a normal infection (1, 2, 6, 7). An HPV-positive lesion progresses to cancer only when the normal control of HPV infection has been lost, and HPV-driven cancers generally represent non-productive infections (8).

## HIGH-RISK HPV E6 AND E7 DRIVE CARCINOGENESIS

Several of the proteins encoded by the early region of the genome are essential for virus persistence and replication (1). The E1 and E2 proteins are required for viral genome replication in both basal and suprabasal cells (9). The E6 and E7 proteins alter signaling in host cell pathways to establish a cellular environment conducive to persistent infection in basal cells and virus replication in terminally differentiated cells (10). The high-risk HPV E5 proteins may subvert protein tyrosine kinase signaling and/or help limit detection of infected cells by the host’s immune system (11). However, it is clear that high-risk HPV E6 and E7 proteins are the major HPV-encoded cancer drivers since their expression is regularly maintained in cancer cells (12–14). High-risk HPV E6 and E7 together are sufficient to immortalize primary human keratinocytes in culture (15, 16) and to induce the genomic instability that is a hallmark of HPV-positive cancers (17, 18). HPV-positive cancer cells remain addicted to the expression of E6 and/or E7 and senesce or die upon inactivation of the HPV oncogenes (19). The essential nature of E6 and E7 in driving carcinogenesis has prompted extensive research on their biological activities and host cell targets. Neither E6 nor E7 has enzymatic activity, but each one binds to host cellular proteins to reprogram or re-direct host cellular enzymes to new substrates (20, 21).

Many investigators (including us) have aimed to identify host cell binding partners of E6 and E7 and to determine the biological consequences of specific interactions between the viral proteins and host cellular proteins. These efforts, coupled with technological advances, have generated long lists of cellular proteins targeted by HPV E6 and E7 and have prompted research questions that could engage the field for many years to come (20, 21). One common view in the field is that E6 and E7 are multifunctional—perhaps even hyperfunctional—proteins and that many of the oncoprotein-host protein interactions are, if not essential, at least important contributors to carcinogenic phenotypes. This would seem to counter the modern view that only a small number of mutations act as drivers in non-viral cancers.

## CANCER IS CAUSED BY A FEW GENETIC CHANGES

In contrast to HPV-associated cancers that are driven by the viral E6 and E7 proteins, the identification of driver mutations in non-viral human cancers has not always started with a smoking gun. Some initial experiments therefore used viral proteins to begin to determine how many distinct drivers were required for human cell transformation in cell-based assay systems. The discovery that certain normal human cell types could undergo oncogenic transformation upon combined introduction of the SV40 large and small tumor (T) antigens, the catalytic subunit of telomerase (TERT), and an oncogenic allele of the Harvey-Ras gene provided some of the earliest evidence that perturbation of a few signaling pathways might be sufficient to enable oncogenic transformation (22, 23).

Advances in sequencing technology and cancer genomics over the next two decades enabled many more efforts to identify the pathways and genes that are drivers in various human cancer types. Collectively, cancer genomics research has established that even though each cancer has accumulated a very large number of genetic and epigenetic lesions, in a given cancer type, there is a characteristic pattern of pathways that are altered by somatic mutation (24). Perhaps most remarkably, the genomics-era studies have arrived at much the same conclusion as the earlier experiments: that most tumors have more than one driver mutation but that among all the many mutations, there are perhaps only four to five driver mutations per cancer (25). Driver mutations in certain genes, for example, *TP53* and *RB1*, which encode the p53 and pRB tumor suppressor proteins, and a few others are mutated in a wide range of cancer types, whereas other driver gene mutations are specific to one or a few cancers (26). Although driver mutations tend to occur predominantly in coding regions, a notable exception is that there is a high frequency of activating mutations in the *TERT* promoter (27), highlighting the importance of telomerase activation in human cancer. The discovery of essential cancer drivers is an important area of research that can enable the characterization of carcinogenic mechanisms and help to identify signaling pathways that may be targets for therapeutic intervention (28).

## THE PARADOX

Should the concept that relatively few genetic insults drive the initiation and progression of most human cancers now be used to advance our understanding of the essential determinants of HPV carcinogenesis? While we know the drivers, E6 and E7, these two proteins are multifunctional, and a dizzying number of cellular interactors have been and are being reported for each of the two proteins (20, 21). If all of them were important determinants of HPV carcinogenesis, it would challenge the paradigm that cancers are driven by a small number of driver mutations. A more likely hypothesis is that only a small number of the signaling pathways regulated by the cellular targets of E6 and E7 act as the drivers for HPV carcinogenesis. Fundamentally, even though the drivers of HPV-positive cancers are well known, we do not know how these drivers drive the tumors. We believe that identifying the essential mediators of the carcinogenic activities of E6 and E7 is critical in the quest to understand mechanisms of HPV-mediated transformation and to identify targets for the development of anti-HPV therapies. In this article, we highlight targets that are well established as essential contributors to HPV transformation and outline the findings that support that this small subset of targets are critical drivers of HPV carcinogenic activity. We acknowledge the likely important roles of many other targets of HPV E6 and E7 in various aspects of the virus life cycle, and we are intrigued by the possibility that if interactions with known targets of E6 and E7 are insufficient to explain the oncoproteins’ carcinogenic activity; other important targets may remain to be discovered.

## WHERE TO START: E6, E7, OR BOTH?

**Fig 1:**
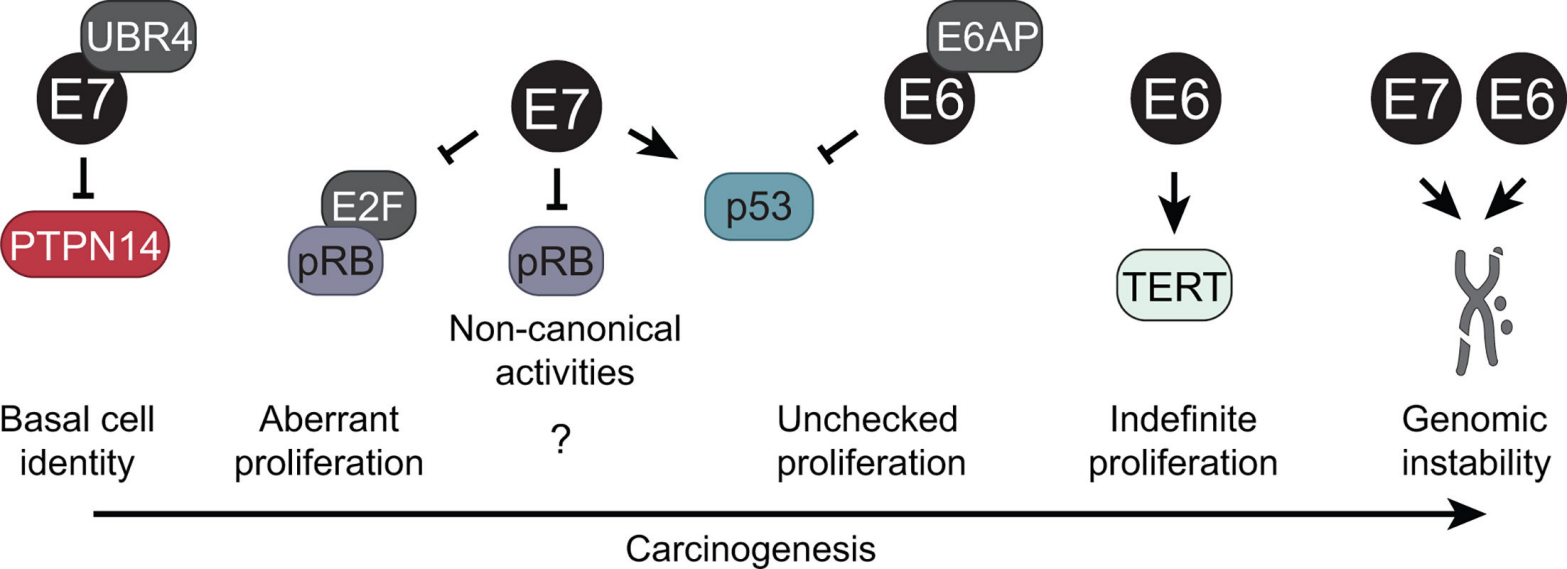
Carcinogenic activities of high-risk HPV E6 and E7. Several activities of the high-risk HPV E6 and E7 oncoproteins are well-established contributors to carcinogenesis. Diverse HPV E7 proteins recruit the ubiquitin ligase UBR4 to degrade the tumor suppressor PTPN14, activating the YAP1 oncoprotein and increasing the propensity of keratinocytes to adopt a basal cell identity. High-risk HPV E7 proteins bind and degrade the retinoblastoma tumor suppressor (pRB), which activates E2F-dependent transcription and drives aberrant proliferation. pRB degradation is also likely to inhibit non-canonical (E2F-independent) activities of pRB. High-risk HPV E7 proteins also stabilize the tumor suppressor p53. Increased p53 levels sensitize cells to p53-dependent cell death that is inhibited by the activity of high-risk HPV E6 proteins, which recruit the ubiquitin ligase E6AP to target p53 for proteasome-mediated degradation, thereby promoting unchecked keratinocyte proliferation. High-risk HPV E6 proteins also increase transcription of the catalytic subunit of human telomerase (TERT), enabling indefinite keratinocyte proliferation. High-risk HPV E6 and E7 proteins can each increase genomic instability in virus-infected cells through multiple mechanisms. The working model of HPV-induced carcinogenic progression is that these events co-operate to enable immortalization and subsequent transformation of high-risk HPV-infected cells.

Any attempt to identify the minimal number of targets of the HPV oncoproteins should first acknowledge their wide-ranging impacts on host cellular processes (29). The high-risk HPV E6 and E7 proteins have unique biological activities that so potently reprogram host signaling that they cause aberrant cell growth and differentiation, extend cellular lifespan, cause genomic instability, and drive carcinogenic progression (1, 2, 30) (Fig. 1). These potent cancer-causing activities are different from those of the “low-risk” HPV E6 and E7 proteins, which cause benign mucosal lesions that very rarely progress to cancer (31).

Experimental data that show high-risk HPV E7 can immortalize primary human keratinocytes, promote genomic instability, and drive tumor formation in *in vivo* models all support the hypothesis that the E7 protein functions as a major oncogenic driver (32). High-risk HPV E6 contributes to many carcinogenic phenotypes and increases the efficiency of E7-mediated keratinocyte immortalization through telomerase activation (33–35). The biological properties of E6 and E7 and the relationship between the two have been established over time in many cell-based experimental assays. Further support for the idea that high-risk E7 is an essential driver of HPV-associated cancers comes from large-scale sequencing data from over 5,000 HPV16-positive patient lesions. In those samples, HPV-positive lesions with a cervical intraepithelial neoplasia (CIN) grade of 3 or higher contained vastly fewer coding variants of HPV16 E7 than of E6, emphasizing that E7 is the major driver of HPV carcinogenesis (36).

Several of the features that distinguish high-risk from low-risk HPV E6 and E7 are well characterized. The expression of a high-risk HPV E7 protein is sensed as an oncogenic stress signal, which triggers a cellular defense response that results in the activation of p53 and expression of the p16^INK4A^ tumor suppressors (37, 38). High-risk HPVs have evolved activities that mute these cellular defense responses. Although both high- and low-risk HPV E7 proteins bind to the retinoblastoma tumor suppressor, pRB, thereby releasing E2F transcription factors, high-risk HPV E7 proteins also target pRB for degradation to blunt the p16^INK4A^-induced senescence response (38). In addition, high-risk, but not low-risk, HPV E6 proteins target the p53 tumor suppressor for proteasomal degradation (39, 40). Normal human cells that express high-risk HPV E7 alone are prone to undergoing p53-dependent cell death, which is no longer observed upon co-expression of high-risk HPV E6 (41). Lastly, high-risk HPV E6 proteins also stimulate telomerase activity (33–35) (Fig. 1).

## BEYOND pRB AND p53

The discovery that the E7/pRB and E6/p53 interactions were critical for HPV transformation was quickly followed by the realization that each of the oncoproteins did more to contribute to carcinogenesis than to inactivate a single tumor suppressor. With respect to high-risk HPV E6, the viral oncoprotein activates telomerase in keratinocytes by increasing transcription of the catalytic subunit of human telomerase (TERT), which is critical for HPV-mediated keratinocyte immortalization (34, 35, 42, 43). The high-risk HPV E6 proteins also bind to PDZ-containing cellular proteins via a conserved PDZ-binding motif at the E6 C-terminus (44), although the biological consequences of these interactions are unknown.

For E7, it is well established that pRB inactivation is necessary but insufficient for E7-mediated transformation (45–55). Two segments of the HPV E7 amino terminus, conserved regions 1 and 2 (CR1 and CR2), are named based on their homology to sequences in the adenovirus E1A protein (56). CR2 of HPV E7 contains the amino acid sequence LxCxE, which is highly conserved across many HPV genotypes and enables E7 to bind pRB and the related pocket proteins RBL1/p130 and RBL2/p107 (57–59). Many mutant forms of E7 have been characterized, and there are multiple versions of high-risk HPV E7 that contain an intact LxCxE motif and can bind pRB but are transformation defective (45–55). Often these forms of E7 carry mutations in the N-terminal CR1 region or in the structured E7 C-terminus, emphasizing the potential importance of other E7-binding partners in transformation.

These and other observations related to the E7/pRB interaction have prompted many targeted or unbiased experiments that collectively have identified a very large number of additional candidate cellular targets of E7 (21). Mass spectrometry-based proteomic analyses determined that the ubiquitin ligase UBR4 (p600) binds to amino terminal sequences of many papillomavirus E7 proteins, and further experiments showed that E7 mutants that are UBR4 binding deficient are also impaired for cellular transformation (52, 60, 61). One substrate of the E7/UBR4 complex is now understood to be protein tyrosine phosphatase non-receptor type 14 (PTPN14). E7, PTPN14, and UBR4 co-purify in gel filtration experiments, and UBR4 is required for the proteasome-mediated degradation of PTPN14 in cells that express HPV E7 (62, 63). E7 recruits UBR4 to target PTPN14 for degradation in a complex distinct from the E7/pRB complex. Many of the mutations in E7 that interfere with its activity in transformation assays overlap with what are now understood to be the binding sites for UBR4 (in the E7 N-terminus) and for PTPN14 (in the E7 C-terminus).

PTPN14 is an inhibitor of YAP1, a potent oncoprotein. YAP1 is frequently amplified, or one of its suppressors is inactivated in many human cancers, and PTPN14 is frequently inactivated in basal cell carcinoma (64, 65). YAP1 is a transcriptional co-regulator that does not bind DNA directly but controls gene expression by binding to TEAD1–4 and other transcription factors. Active YAP1 is hypophosphorylated and localized to the nucleus, where YAP1/TEAD-dependent transcription promotes cell stemness and self-renewal and impairs differentiation (66, 67). Like high-risk HPV E7 proteins mutated in the LxCxE motif, E7 proteins that are mutated in the N-terminal UBR4-binding residue or the C-terminal arginine necessary for PTPN14 binding are defective for extending the lifespan of primary human keratinocytes (48, 50, 68–70). The lifespan extension defect of a C-terminal arginine mutant is rescued by PTPN14 knockout (69).

Other review articles can provide long lists of the many host cell proteins that have been reported to bind to HPV E6 or E7 (20, 21). However, for the interactions highlighted so far, the structural basis of the interaction is known, and/or the interaction depends on amino acids that are highly conserved in E6 or E7. For E7/pRB and E7/PTPN14, E7 binds directly to the host cellular protein, and a crystal structure of the full- or partial-length proteins in complex with one another has been solved (71, 72). In each case, the interaction depends on amino acids in E7 that are highly conserved across the HPV phylogeny. Although the structure of UBR4 in complex with E7 has not been solved, the interaction requires at least one highly conserved acidic amino acid in the E7 N-terminal domain (52, 61, 62, 68). Decades of research also determined how E6 binds to p53. High-risk HPV E6 first engages the amino acid sequence LxxLL present in the ubiquitin ligase E6AP. Upon binding to E6AP, E6 adopts a conformation that can bind to p53 (73, 74). Independent of the interaction with E6AP, a short amino acid sequence that is present at the carboxyl terminus of high-risk, but not low-risk, E6 proteins enables binding of high-risk HPV E6 to cellular proteins that contain PDZ motifs (44).

Finally, each of these conserved activities of the viral oncoproteins contributes to processes required during normal virus infection. Inactivation of pRB contributes to the E2F-mediated expression of the DNA replication machinery that is required for virus genome replication in differentiated suprabasal epithelial cells (10). p53 degradation inhibits the cytostatic or cytotoxic signals that would otherwise result from the expression of high-risk HPV E7 (41). Similarly, E7 expression triggers a p16^INK4A^-mediated senescence signal that is overcome by pRB degradation (38). PTPN14 degradation enables E7 to activate the YAP1 oncoprotein and is proposed to promote the retention of HPV-infected cells in the basal layer of stratified epithelial tissue (68, 69, 75).

## ARE THERE MINIMAL DETERMINANTS OF HPV CARCINOGENIC ACTIVITY?

**Fig 2:**
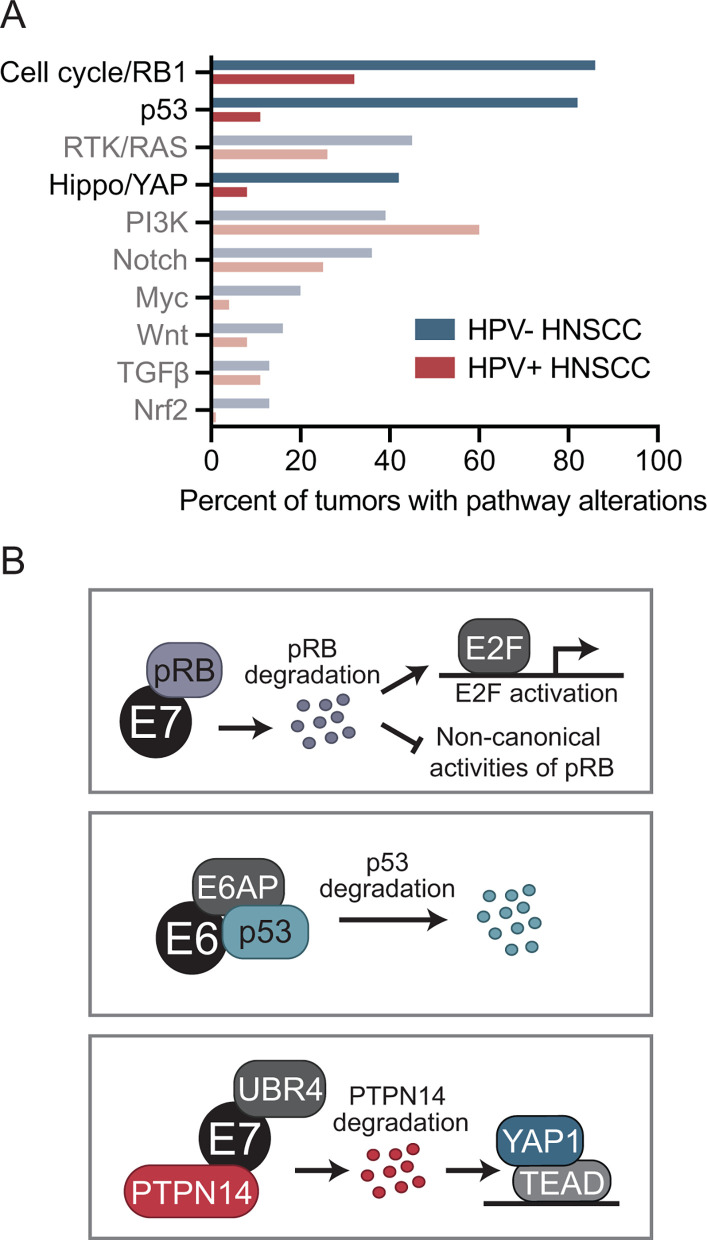
Proposed drivers of HPV carcinogenesis. (**A**) Mutation rates in growth-promoting signaling pathways in head and neck squamous cell carcinoma. Data from reference (24) were re-graphed to summarize somatic mutation rate data for genes in 10 different growth-promoting pathways in HPV-positive (HPV+) and HPV-negative (HPV−) HNSCC. Values indicate the percent of samples of each tumor type that have at least one genomic alteration in a signaling pathway. See reference (24) for more detail. (**B**) Conserved activities of high-risk HPV E7 oncoproteins. High-risk HPV oncoproteins bind and degrade pRB and, in a separate complex, recruit UBR4 to degrade PTPN14. High-risk HPV E6 proteins recruit the ubiquitin ligase E6AP to target p53 for proteasome-mediated degradation. Other host cell targets of HPV oncoproteins may contribute to cellular reprogramming that is required for virus replication, contribute to their carcinogenic activity, or both.

Based on these results, it is intriguing to hypothesize that the combined inactivation of pRB, p53, and PTPN14, together with TERT activation, accounts for most or all of the carcinogenic activity of the HPV oncoproteins. Robust support for such a hypothesis should draw on more data than those generated through protein-protein interaction studies alone. As for non-viral cancers, there is now a wealth of genomic data from HPV-induced cancers, and somatic mutation data can provide important information regarding cancer drivers. It is likely that an essential HPV-targeted oncogenic pathway is more frequently mutated in HPV-negative cancers than in HPV-positive ones. Initial observations showed that in a relatively small number of HPV-positive cancers or cancer cell lines, there were not gross alterations in the p53 or pRB tumor suppressor pathways (76, 77). A more recent analysis of many more cervical cancers, which are nearly all HPV positive, and head and neck squamous cell carcinomas (HNSCCs), which can be HPV positive or negative, extended this finding (78). In that analysis, mutations in *TP53, RB1,* and *TERT* were almost completely absent in HPV-positive cancers but were common in HPV-negative ones. Finally, data from the Cancer Genome Atlas provide insight into mutational patterns in additional growth-promoting pathways (24, 75). In that data set, the three mitogenic pathways that are most differentially altered with high mutation rates in HPV-negative and low mutation rates in HPV-positive HNSCC are p53, cell cycle/pRB, and the Hippo pathway that regulates YAP (Fig. 2A). Other mitogenic pathways do not exhibit the same pattern of differential mutation in HPV-positive vs HPV-negative HNSCCs (24). These data are entirely consistent with the idea that the combined inactivation of p53 by E6, and pRB and PTPN14 by E7, plus telomerase activity, may be essential determinants of HPV carcinogenesis (Fig. 2B). If so, it would be reasonable to infer that the major transforming activity of E7 is based on inactivating pRB and activating YAP1.

A complementary approach to test the hypothesis about host cell drivers downstream of HPV E7 is to use well-characterized E7 mutants and genome-wide transcriptional data. Disruption of a cellular signaling pathway causes characteristic changes to host cell transcription, and the large-scale changes in gene expression induced by HPV E7 therefore reflect its effects on specific cellular pathways (68, 79). If inactivating pRB and degrading PTPN14 were the major biological activities of a high-risk HPV E7, then the transcriptional alterations it induces would mostly reflect pRB and PTPN14 inactivation. pRB inactivation causes several large-scale changes to host transcription (80, 81). pRB suppresses cell cycle progression by binding to E2F transcription factors and converting them to repressors. Inactivation of pRB via mutation or the activity of a viral oncoprotein therefore activates E2F-regulated promoters in the G1 phase of the cell cycle (82). Beyond its activity at E2F-dependent promoters, recent ChIP-seq experiments have revealed that pRB binds to enhancers and regulates cell type-specific gene expression during later phases of the cell cycle through E2F-independent mechanisms (83).

Some of our previously published data can help to determine the contributions of PTPN14 and pRB inactivation to the transcriptional alterations caused by HPV16 E7 expression. We characterized a mutant of HPV16 E7 that cannot bind UBR4 or degrade PTPN14 and compared it to an HPV16 E7 mutant that cannot bind pRB, generating gene expression data from primary human keratinocytes that express one of the mutants, or wild-type HPV16 E7 (68). When HPV16 E7 cannot degrade PTPN14, it causes changes in gene expression that resemble those induced by wild-type HPV16 E7, with a major difference being that the mutant is unable to repress the expression of certain markers of epithelial differentiation. When HPV16 E7 cannot degrade pRB, its residual effect on gene expression resembles the effect of PTPN14 knockout. Both observations suggest that the transcriptional signature of HPV16 E7 is dominated by PTPN14 degradation and pRB degradation.

## WHY DOES IT MATTER, AND WHAT ABOUT ALL THE OTHER INTERACTORS?

Viral oncoproteins are well established as multifunctional proteins that rewire numerous host cell signaling pathways, and our goal is not to dispute the idea that HPV oncoproteins have multiple targets that play many roles in virus biology. However, if it is true that, like for non-viral cancers, HPV-associated cancers are driven by just a handful of targets, it may be useful to consider what roles are played by the many other binding partners of HPV E6 and E7 in virus-infected cells. There is no doubt that many of these host cell proteins are necessary to facilitate certain aspects of the viral life cycle. For instance, certain host targets of HPV E6 and E7 could be important in immune evasion, in establishing tropism for epithelial cells at specific anatomical sites, or in other aspects of host cell reprogramming unique to certain HPV genotypes (20, 21).

It should also be acknowledged that p53, pRB, and YAP1 are at central nodes in host cell signaling pathways (84–86). Therefore, some of the previously documented effects of E6 and/or E7 may be downstream of one or more of these nodes. Consistent with this idea, expression of the high-risk HPV oncoproteins causes cells to exhibit many phenotypes characteristic of the hallmarks of cancer (1, 2, 6, 29). It may be informative to consider whether some or all of these phenotypes are the result of the virus’ effects on one or more of pRB, YAP1, p53, and TERT. For example, the high-risk HPV oncoproteins have a well-documented ability to induce genomic instability by increasing the expression of apolipoprotein B mRNA-editing enzyme, catalytic polypeptide (APOBEC) family members and by interfering with faithful repair of double-strand DNA breaks (87, 88), and experiments to test which host cell target(s) of E6 and E7 enable these activities could be informative. Identifying which targets and which pathways are critical for HPV carcinogenesis will not only provide important new information related to host cell biology but has important potential therapeutic impact. Several of the pathways here may be druggable. Small molecule inhibitors of YAP1 are in development (89). Cells that lack pRB activity are dependent on Skp2 for survival (90), and there are small molecule inhibitors of Skp2 (91). Mechanistic understanding of how an HPV oncoprotein acts on one host cell protein, such as PTPN14, to control signaling through a central node, such as YAP1, may further help to refine the specificity of therapeutics that target host proteins.

Further efforts to study host cell targets of E6 and E7 should also consider certain limitations of the many HPV-host interaction studies in the literature. These experiments are powerful discovery tools but have nonetheless been conducted in many cell types, with HPV E6 and E7 from a variety of virus genotypes, under conditions that can result in a wide range of E6 or E7 expression levels (20, 21). Researchers should therefore be mindful of the ideas that certain HPV oncoprotein-host cell interactions may be detectable only in specific experimental settings and that not every interaction that can be observed may contribute directly to HPV infection or disease. We do not discount the impact of the many efforts to identify HPV-host protein interactions, which come from many laboratories, including from ours. They have collectively enabled important advances in our field and have advanced the mechanistic understanding of many normal cellular processes.

## CONCLUDING REMARKS

In considering the minimal determinants of HPV carcinogenesis, our goal is not to be restrictive but to promote new discussions and encourage new experimental approaches. Continued progress in HPV research should draw upon the wealth of data on virus-host interactions that come from many years of impactful research but should also benefit from new principles revealed by current studies on the drivers of non-viral cancers. Updating our thinking in this way could be transformative. Even though HPV-mediated carcinogenesis is entirely dependent on the expression of the viral oncogenes, our understanding of how HPV E6 and E7 drive carcinogenesis remains incomplete, and therapeutic inhibition of HPV E6 and/or E7 has been unsuccessful so far. The concept that the oncogenic activities of the viral E6 and E7 proteins are, to a large part, due to their ability to bind host cellular proteins is well established. The lack of enzymatic activity associated with either protein and the fact that several of their host cell targets are at essential nodes in cell pathways mean that developing specific and non-toxic HPV antivirals is challenging. Efforts to develop or identify drugs that can treat HPV disease could benefit tremendously from the ability to focus therapeutic discovery efforts on a few essential targets of HPV oncoproteins. We also propose that similar approaches could be used to define essential drivers of other viral cancers. These approaches include the analysis of cancer genomic data from virus-positive and virus-negative cancers and the analysis of the transcriptional aberrations caused by the expression of viral oncoproteins in normal human cells.
